# The Efficacy and Tolerability of Colistin Versus Non-Colistin Antimicrobial Regimens Among Hospitalized COVID-19 Patients with Multidrug-Resistant Bacterial Superinfection: An Observational Multicenter Study

**DOI:** 10.3390/medicina61050884

**Published:** 2025-05-13

**Authors:** Alzahraa M. Fahmy, Marwa O. Elgendy, Alaa Aboud Mohamed, Mohamed S. Imam, Abdullah Nasser Alharbi, Muhammad Husayn Al-Anezi, Omar Mana Aldhafeeri, Saif Mamdouh Aldhafeeri, Jawaher A. Ajeebi, Marwa Kamal, Hasnaa Osama

**Affiliations:** 1 Tropical Medicine and Infectious Diseases Department, Faculty of Medicine, Beni-Suef University, Beni-Suef 62511, Egypt; alzhraamohamed@med.bsu.edu.eg; 2 Department of Clinical Pharmacy, Beni-Suef University Hospitals, Faculty of Medicine, Beni-Suef University, Beni-Suef 62511, Egypt; 3 Department of Clinical Pharmacy, Faculty of Pharmacy, Nahda University (NUB), Beni-Suef 62511, Egypt; 4 Department of Tropical Medicine, Faculty of Medicine, Beni-Suef University, Beni-Suef 62511, Egypt; dralaaboud2005@yahoo.com; 5 Department of Clinical Pharmacy, National Cancer Institute, Cairo University, Fom El Khalig Square, Kasr Al-Aini Street, Cairo 11796, Egypt; 6 College of Pharmacy, Hafr Al Batin University, Hafr Al-Batin 31991, Saudi Arabia; abdullah.aldugaim@outlook.sa (A.N.A.); omarmani029@gmail.com (O.M.A.); callofnow@gmail.com (S.M.A.); 7 College of Pharmacy, Jazan University, Jazan 82912, Saudi Arabia; jawaher.ajeebi@gmail.com; 8 Department of Clinical Pharmacy, Faculty of Pharmacy, Fayoum University, Fayoum 63514, Egypt; marwa.tolba@yahoo.com; 9 Clinical Pharmacy Department, Faculty of Pharmacy, Beni-Suef University, Beni-Suef 62511, Egypt; hasnaa_osama2010@yahoo.com

**Keywords:** colistin, COVID-19, Gram-negative bacteria, secondary infection

## Abstract

*Background and Objectives*: Bacterial infections amongst COVID-19 patients could be associated with worsened outcomes. This study aimed to investigate the efficacy of colistin antibiotic in multidrug-resistant (MDR) Gram-negative (-ve) secondary bacterial infections among hospitalized COVID-19 patients. *Materials and Methods*: In this multicentered retrospective study, we analyzed data from the medical records of 116 patients diagnosed with COVID-19 infection and secondary Gram-negative MDR bacterial respiratory infections. *Results*: We compared those assigned to colistin versus non-colistin-based antimicrobial therapy. The two arms of the study were similar in baseline clinical features, demographics, and Gram-negative pathogens’ distribution. Acinetobacter baumannii (51.7%) was the major pathogen, followed by Klebsiella pneumonia (26.7%). Patients who received colistin-based antimicrobial regimen showed a non-significant difference compared to non-colistin antimicrobial (NCA) therapy (*p* > 0.05) in the main outcomes. Nephrotoxicity was significantly higher in the IV colistin group, compared to the control (34.1% and 15.3%, *p* = 0.018). There were substantial differences observed in the levels of serum creatinine and urea among the study arms (*p* = 0.029 and <0.001, respectively). *Conclusions*: The combination of colistin with other antimicrobial agents showed comparable results to that of NCA regimens in hospitalized COVID-19 patients with superinfections with multidrug-resistant bacterial isolates; however, there was a notably elevated incidence of nephrotoxicity with colistin antimicrobial therapy. Further randomized controlled trials are needed to assess the therapeutic benefits and tolerability of colistin antimicrobial therapy.

## 1. Introduction

Since the emergence of the COVID-19 outbreak, the majority of efforts has been directed to the management of the virus [1,2,3]. Several emerging variants have been isolated with substantial variability in disease severity, evasion of the immune system, and response to therapeutics [4,5,6]. According to the World Health Organization (WHO), five variants of SARS-CoV-2 are designated as variants of concern with higher rates of transmission and relatively severe illnesses [7,8], and they warrant close monitoring [9,10].

Many studies reported respiratory bacterial superinfection among patients with COVID-19, with variable prevalence across the globe, especially among hospitalized patients. Severe cases of COVID-19 infections have much lower CD4 and CD8 T cells, which is conducive to higher vulnerability to bacterial coinfection and, consequently, higher fatality [11,12].

Bacterial infection transmission occurs within hospitals [13], either directly or indirectly, among hospitalized patients through contact with workers in healthcare settings and hospital equipment, such as ventilators and catheters [14]. Several isolates are well linked with nosocomial infections, such as Acinetobacter species, Klebsiella, Pseudomonas, and Staphylococcus species [15,16].

There is evidence indicating that the presence of bacterial pathogens as secondary infections can exacerbate the clinical prognosis of individuals with COVID-19 [17,18]. Bacterial infections acquired during a hospital stay are likely resistant to multiple antimicrobial drugs, resulting in a considerable challenge in managing COVID-19 patients [19,20].

Colistin is an antibacterial agent that has been used for several decades [21,22]. However, it was withdrawn due to safety concerns, including its neurotoxicity and nephrotoxicity [23,24]. Recently, because of the global concern about widespread nosocomial infections with carbapenem-resistant bacterial isolates, colistin has re-emerged [25,26].

The emergence of antibiotic resistance through the COVID-19 outbreak is significantly linked to the prevalence of Klebsiella pneumoniae, particularly carbapenemase-producing strains. The prior report indicated that the incidence of carbapenem-resistant K. pneumoniae (CRKP) escalated during the initial wave of the pandemic [27]. Numerous studies have indicated that the incidence of CRKP infections escalated during the pandemic. Currently, colistin in combination with other antimicrobial agents, such as meropenem or tigecycline, has been considered the cornerstone therapy for Gram-negative resistant isolates despite its considerable adverse effects [22,28]. Although colistin use has considerably increased in clinical practice, no standardized consensus has been developed for the optimum dose or route of administration.

Therefore, the main purpose of this study was to evaluate the tolerance and effectiveness of colistin-based treatment regimens for MDR Gram-negative bacterial superinfection in hospitalized patients with COVID-19.

## 2. Results

### 2.1. Baseline Study Population Characteristics

**Figure 1:**
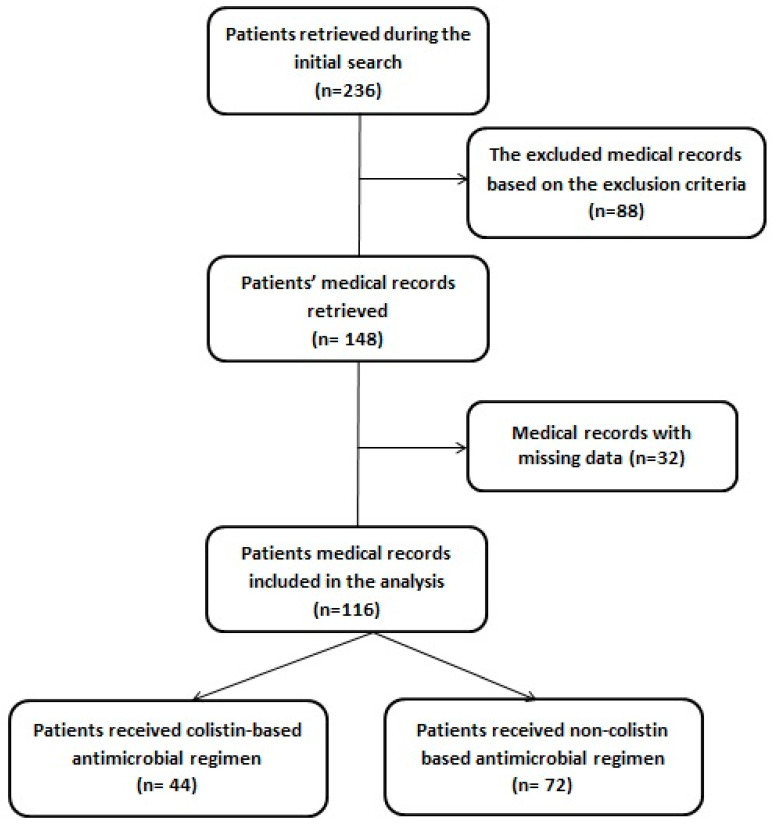
Flowchart of the study.

In total, 116 COVID-19-confirmed patients using PCR met the inclusion criteria and were included in the analysis. Out of the total, 44 (37.9%) patients received IV colistin-based regimen, and 72 (62.1%) received NCA regimen. Figure 1 summarizes the study’s flowchart.

The demographics, clinical characteristics, and other therapeutic regimens of antimicrobials or specific drugs for COVID-19 of patients are illustrated in Table 1. The mean ± SD age was 57.9 ± 8.7 years and ranged between 46 and 79 years. Males made up 49.1% of the overall study population. The difference in baseline demographic data and comorbidities upon admission of patients in the study groups was statistically non-significant. In the two arms of the study, the combination with antimicrobial therapy was non-significantly different. Carbapenem antibiotics were commonly prescribed in both colistin (47.7%) and non-colistin (40.3%) groups.

**Figure 2:**
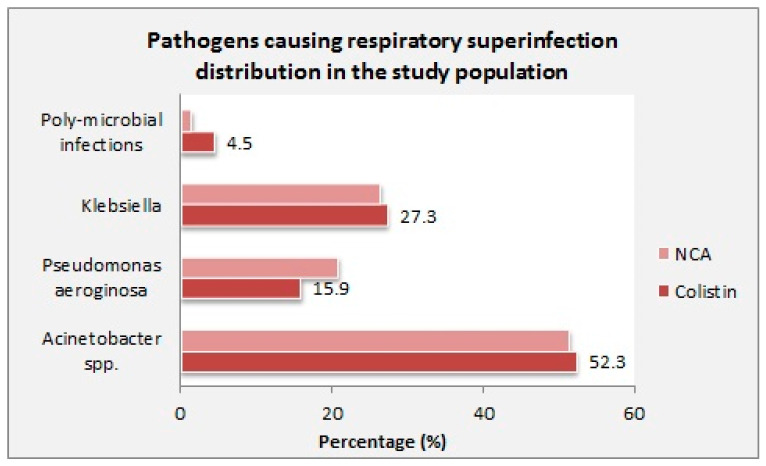
Microbial distribution in the study population. NCA, non-colistin antimicrobial regimen.

Of all included patients, co-infections with Klebsiella pneumonia, Acinetobacter baumannii, and Pseudomonas aeruginosa infections were the most common bacterial isolates, with a non-significant difference in the distribution of microbial isolates between the study groups (*p* > 0.05) (Figure 2).

### 2.2. Assessment of Efficacy

The use of the colistin-based regimen was linked with a reduced length of hospitalization, with a mean ± SD of 11.3 ± 3.8 days, compared to the non-colistin group (12.8 ± 4.7); however, the difference was non-significant (*p* = 0.073). The number of patients assigned for mechanical ventilation did not differ significantly between the study groups: 22.7% and and 23.6%, *p*-value = 0.913, for colistin and non-colistin based regimens, respectively. The outcomes of the study groups are presented in Table 2. In the same context, a non-significant difference was observed in the other outcomes: the need for admission to the ICU, the duration of the ICU stay, and the all-cause mortality at 30 days between the two study arms. The colistin group showed a higher microbiological eradication rate (n = 35, 79.5%) than that observed with non-colistin-based antibiotic regimen (n = 52, 72.2%); however, the difference did not reach significance (*p* = 0.377).

### 2.3. Assessment of Systemic Toxicity

Of the overall included patients, 13 (11.2%) showed elevations in ALT and AST serum levels with treatment. Of them, eight (18.2%) were receiving IV colistin-based regimens, and the other five (6.9%) patients were in the NCA group. However, no significant difference was observed between the study groups (*p* = 0.063).

Nephrotoxicity incidence was higher in the colistin group compared to the NCA group (34.1% vs. 15.3%, *p* = 0.018). Among the 44 patients who received IV colistin, risk and injury KDIGO categories occurred in 8 (18.2%) and 5 (11.4%), respectively, and failure was observed in 2 (4.5%) patients who required discontinuation of treatment. Eleven (15.3%) patients in the control group had nephrotoxicity in the risk category of KDIGO.

**Figure 3:**
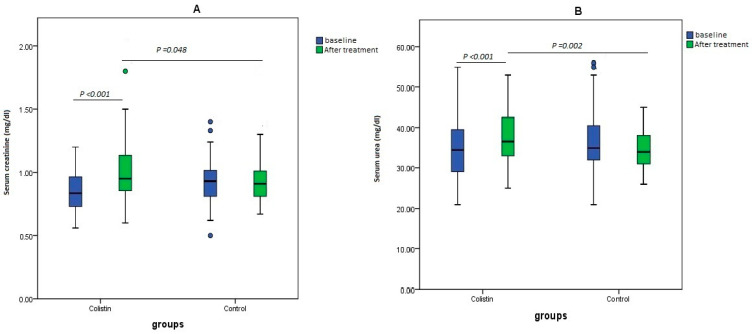
The changes in (**A**) serum creatinine (mg/dL) and (**B**) serum urea (mg/dL) levels in the study groups.

At baseline, the difference in creatinine levels between the study population was non-significant (*p* = 0.071), with an overall mean ± SD of 0.89 ± 0.16 mg/dL. After treatment, the overall difference in serum creatinine levels between the study arms was significant (*p* = 0.048), with a high increase in SCr levels observed with colistin-based regimens and a mean difference (MD) of 0.28 [95%CI; 0.2, 0.39; *p*-value < 0.001], while the control group showed lower change in creatinine levels [MD: 0.04; 95% CI; −0.01, 0.09; *p* = 0.109] (Figure 3A).

The difference in urea levels between the study populations at baseline was non-significant (*p* = 0.217), with an overall mean ± SD of 35.5 ± 8.01 mg/dL. Following the completion of therapy, urea levels increased from baseline with intravenous colistin [MD: 4.1, 95% CI: 2.04 to 6.22, *p* < 0.001]. Furthermore, there was a significant difference between the two arms of the study (*p* = 0.002) (Figure 3B).

To determine the risk factors associated with nephrotoxicity as defined by KDIGO criteria in both colistin- and NCA-treated cohort arms, logistic regression analyses were performed. The outcomes showed that patients receiving colistin experienced significantly higher nephrotoxicity (OR: 2.86; 95%CI: 1.17 to 7.02, *p* = 0.021). In other words, the incidence of AKI in the colistin group was 2.86 times higher than that experienced in non-colistin based regimens. Other factors significantly associated with nephrotoxicity were age older than 60 years (OR: 1.08; 95%CI: 1.012 to 1.13; *p* value  =  0.017), diabetes mellitus (OR: 2.65; 95% CI, 1.07 to 6.51, *p* = 0.034), remdesivir use (OR: 2.93; 95%CI: 1.15 to 7.463, *p* = 0.024), and tocilizumab use (OR: 2.57; 95% CI, 1.02 to 6.46; *p* = 0.045) (Supplementary Table S1).

Multivariate logistic regression analysis showed a significant association between chronic respiratory diseases, nephrotoxicity, and the incidence of mortality at 30 days, with odds ratios (ORs) of 1.9 [95% CI; 1.02, 3.9] and [OR: 2.6, 95% CI: 1.1, 6.2], respectively (Table 3).

## 3. Discussion

The primary objective of this study was to examine the clinical outcomes and tolerability of using a colistin-based regimen of antimicrobial therapy in the treatment of hospitalized patients with COVID-19 who have developed MDR bacterial superinfection. The main finding of this study is that the colistin-based antimicrobial regimen did not show superiority compared to the other antimicrobial regimens. Moreover, nephrotoxicity was significantly higher with colistin-based antimicrobial therapy among COVID-19 patients with MDR Gram-negative respiratory superinfection.

The significance of secondary bacterial infection, especially with Gram-negative pathogens, is well recognized by many researchers [29,30]. Many studies investigated the prevalence of microbiological pathogens in the respiratory tract specimens of adult COVID-19 cases admitted to hospitals [31]. Most of these studies reported that K. pneumoniae, A. baumannii, and Pseudomonas species were the most commonly isolated Gram-negative agents from patients’ specimens [32], as were S. aureus for Gram-positive bacteria [33,34]. In this study, Acinetobacter baumannii (51.7%) was found to be the most common Gram-negative microorganism, followed by K. pneumonia (26.7%) and P. aeruginosa (18.97%).

Colistin, a polymyxin E antibiotic, gradually restored its popularity because of its potential efficacy against infections that result from multidrug-resistant (MDR) pathogens [35,36]. However, only limited examinations of its efficacy and tolerability are available for COVID-19 patients with secondary MDR bacterial infections [37,38].

Lim et al. assessed the effects of different combinations against MDR Gram-negative bacterial infections and observed non-significant differences in mortality rates among cases treated with colistin and NCA arms (35.5% and 38.5%, respectively, *p* = 0.80) [39]. The same was also reported by Balkan et al., thus conforming to our findings. In a prospective trial, including patients assigned to colistin versus comparators, including meropenem or imipenem or ampicillin–sulbactam, the administration of beta-lactams antimicrobial therapy was found to be linked with lower overall mortality [40]. The same was observed in our results, where higher mortality was associated with colistin-based regimens; however, the effect did not reach significance.

A previous study reported that four bacterial species were predominantly found in COVID-19 patients: Klebsiella pneumoniae, Acinetobacter baumannii, Pseudomonas aeruginosa, and *Escherichia coli*. Among these, K. pneumoniae* and *A. baumannii* showed the highest rates of co-infection with SARS-CoV-2, which was associated with increased mortality in this study. The colistin showed highly efficacy against Pseudomonas aeruginosa and Klebsiella pneumonia [41,42].

The regression analysis of our results revealed nephrotoxicity and chronic pulmonary disease as the two important factors highly linked with mortality among our study population. Indeed, these results are consistent with several studies which identified acute kidney injury and pulmonary diseases as predictors of in-hospital mortality [43,44]. The administration of colistin-based antimicrobial therapy showed a higher microbiological eradication rate than that observed with the non-colistin-based antibiotic regimen, with non-significant difference between groups (79.5% vs. 72.2%, *p* = 0.377).

It is noteworthy that several factors have been identified in the clinical response to colistin therapy, including IV colistin loading dose, sepsis, nephrotoxicity, gender, and the score of Acute Physiology and Chronic Health Evaluation II [45]. Considering the limited sample size, considerable variation in clinical and demographic attributes within our study cohort, and comorbidity with COVID-19, it might be challenging to detect significant predictive factors for response to colistin therapy.

The infection of COVID-19 mainly affects the lungs; however, the incidence of acute kidney injury (AKI), shown as a notable increase in serum creatinine and urea levels, is also well documented [46]. Replicated studies reported an incidence rate of AKI ranging from 5% to 23% among hospitalized COVID-19 patients and reaching over 60% in severe cases [47,48]. Since colistin is associated with the possibility of nephrotoxicity, the potential synergistic effect of the harmful effects of COVID-19 on the kidney would be a subject of concern. In the current study, the incidence rate of nephrotoxicity appeared to be significantly higher with colistin-based regimens (34.1% vs. 15.3%, *p* = 0.018).

Furthermore, the conducted multivariable regression analyses to assess the robustness of our main findings revealed that colistin-based regimens increased the risk of nephrotoxicity by 2.86 times that of NCA (95%CI: 1.17 to 7.02, *p* = 0.021). Old age (>60 years) and diabetes mellitus comorbidity were consistently associated with nephrotoxicity. Remdesivir and tocilizumab were also identified as risk factors for the incidence of AKI I in the study cohorts. Hence, based on our findings, the risk of nephrotoxicity among COVID-19 patients seems to be multifactorial. These findings are in accordance with those reported by Rocco et al.’s (2013) study, which identified older age and sepsis as strongly associated risk factors for the incidence of AKI with receiving a high dose of colistin methanesulfonate intravenously, either alone or in combination with other potentially nephrotoxic antibiotics [49]. Similarly, numerous studies reported a significant association of remdesivir, tocilizumab, and lopinavir/ritonavir with an increased odds of AKI among COVID-19 patients [50,51].

It is worth noting that we could not observe significant hepatotoxicity with colistin-based regimens; however, a relative elevation in liver enzymes with the IV colistin regimen compared to NCA regimens [8 (18.2%) vs. 5 (6.9%), *p* = 0.063] was detected. These findings were consistent with previous studies [35,52].

Therefore, polymyxin-based antimicrobial regimens might be an adequate alternative option for MDR Gram-negative bacterial respiratory infection, especially in conditions where resistance for β-lactam–β-lactamase inhibitors is present [53]. However, kidney function should be cautiously monitored in such cases. In addition, the inhalation route of colistin can be a less toxic alternative according to several recent studies [36,54,55].

The retrospective design is the major limitation of our investigation. Other limitations encompass the relatively small sample size; the presence of other antimicrobial therapies; and the absence of follow-up for long-term, recurrence, or emergence of resistance assessment. In addition, we could not assess the neurotoxic effects of colistin. Additional randomized controlled studies that use genomic and artificial intelligence techniques like WGCNA and LIME may be able to pinpoint the important genes affecting the toxicity and efficacy of colistin in COVID-19 patients. Examining hub genes, like PGLYRP4 and HEPHL1, that have been connected to COVID-19 may shed light on how colistin affects the immune system. Colistin may improve results and lessen nephrotoxicity when used in conjunction with FDA-approved medications that target certain genes [56].

## 4. Materials and Methods

### 4.1. Ethical Approval

Before proceeding with the trial, the protocol underwent a comprehensive evaluation and received approval from the research ethics committee of Faculty of pharmacy, Beni-Suef University.

### 4.2. Study Design and Target Population

This cohort study was conducted retrospectively at many Egyptian healthcare centers between March 2021 and August 2021. In this study, we compared the tolerability and effectiveness of IV colistin in comparison to non-colistin antimicrobial (NCA) regimens in COVID-19 patients with MDR-secondary Gram-negative bacterial infection. Patients who were treated with colistin or NCA regimens for ≥72 h were identified from medical records. Severe, non-critically ill COVID-19 adult patients aged > 18 years and who had pneumonia and with confirmed MDR Gram-negative bacterial superinfection were eligible for the study. The determination of the severity of the COVID-19 infection was made in accordance with the definitions provided by the World Health Organization (WHO) [57]. The included patients were identified as positive COVID-19 cases using the reverse transcription–polymerase chain reaction (RT-PCR) test. Pneumonia diagnosis in patients was defined either by chest imaging and at least 2 clinical features of pneumonia [58,59].

A bacterial infection was confirmed using microbiological cultures. MDR bacteria were defined as microbial pathogens exhibiting resistance to a minimum of one antibiotic in three or more distinct antibiotic groups [11,60].

The exclusion criteria included patients younger than 18 years old; pregnancy; immunocompromised patients with malignancy or HIV infection; and patients with renal impairment, such as chronic kidney disease (CKD), or assignment to renal-replacement therapy (RRT). Patients’ records that fulfilled the predetermined inclusion criteria were included in the analysis.

We divided the whole cohort into two groups as follows: the intravenous colistin-based antimicrobial regimen (9 million international units (MIU) of colistin in divided doses for 10 days, adjusted according to the renal function), and the control group, which included patients who received systemic antibiotic regimens without colistin. The Sanford guide on antimicrobial therapy was used to determine the adequate dose of antimicrobial therapy for each patient.

### 4.3. Study Outcomes

The following data were extracted from the medical records of the included patients: demographics, medical history, hospital settings, dosage of colistin used, and the co-administered antibiotics. The main outcomes included microbiological eradication rate, defined as the absence of Gram-negative isolates with respiratory cultures at the end of antibiotic therapy, hospital duration of stay, admission to intensive care unit (ICU), ventilation needs, and survival rate of the participants. Laboratory test outcomes such as alanine aminotransferase (ALT), aspartate aminotransferase (AST), D-dimer hemoglobin, platelet count, and C-reactive protein (CRP) were also collected. Serum creatinine (SCr) and urea levels at baseline and post-intervention were recorded to detect antibiotic-associated nephrotoxicity, in accordance with the Kidney Disease Outcomes Quality Initiative (KDOQI) guidelines [61].

Drug-sensitivity tests and sputum cultures were performed according to hospital laboratory standards using the disk diffusion method. The microbiological-susceptibility testing was performed on the causative pathogen to common antibiotics, including carbapenem, amino-glycosides, cephalosporins, piperacillin/tazobactam, fluoroquinolones, colistin, and tigecycline. The breakpoints identified by the Clinical and Laboratory Standards Institute (CLSI) were applied in the susceptibility testing. Susceptibility to colistin was defined as in vitro minimum inhibitory concentration (MIC) < 2 mg/L.

### 4.4. Statistical Analysis

IBM SPSS Statistics version 16 software, created by Install Shield Corporation, Inc., Chicago, IL, USA, was used to perform the descriptive analysis. The Kolmogorov–Smirnov test was performed to determine the normality of the data, and the continuous variables were represented by the mean ± standard deviation (SD). The categorical data were represented in the form of frequencies and percentages. A pairwise comparison utilizing a paired Student’s *t*-test for normally distributed data was employed to evaluate the differences between the research groups. Wilcoxon signed rank was used for paired comparisons with values that were not normally distributed, and the Mann–Whitney U test was used for comparisons between two independent groups. Categorical variables were compared using the Chi-square test. A logistic regression analysis was conducted to investigate the variables that are correlated with death at the 30-day outcome and the independent predictors associated with acute kidney injury in the study population.

## 5. Conclusions

The presence of multidrug-resistant respiratory infections in severe cases of COVID-19 infections is considerably challenging for healthcare professionals. The effectiveness of colistin combined with other antimicrobial therapies showed comparable results to that of non-colistin-based regimens among hospitalized COVID-19 patients with secondary infections with multidrug-resistant bacterial isolates; however, there was a substantial change in the occurrence of nephrotoxicity between the groups with and without colistin antimicrobial therapy. Further robustly designed randomized controlled studies with a larger cohort are necessary to assess the therapeutic benefits of colistin, its safety, and its potential antiviral activity against COVID-19.

## Figures and Tables

**Table 1:** Baseline demographic and clinical characteristics in the study groups.

Parameter	Intravenous Colistin Group (n = 44)	Non-Colistin (Control Group) (n = 72)	*p*-Value
Age	58.9 ± 9.6	57.3 ± 8.2	0.316
Gender (male, %)	18 (40.9%)	39 (54.2)	0.166
Co-administered antibiotic therapy (n, %)			
Carbapenem	21 (47.7)	29 (40.3)	0.432
Piperacillin–tazobactam	12 (27.3)	16 (22.2)	0.537
Macrolides	10 (22.7)	18 (25)	0.781
Cephalosporins	4 (9.1)	11 (15.3)	0.335
Fluoroquinolones	3 (6.8)	7 (9.7)	0.589
Comorbidities (n, %)			
Obesity	12 (27.3)	15 (20.8)	0.426
Chronic respiratory diseases	3 (6.8)	2 (2.8)	0.298
Diabetes mellitus	16 (36.4)	23 (31.9)	0.625
Hypertension	10 (22.7)	13 (18.1)	0.540
Laboratory findings (mean ± SD)			
Hemoglobin (g/dL)	12.6 ± 1.37	12.4 ± 1.34	0.310
Platelet (cells/mm^3^)	349.6 ± 49.7	331.8 ± 69.8	0.132
Lymphocytes (%)	19.3 ± 6.2	18.9 ± 6.7	0.716
Serum urea (mg/dL)	34.3 ± 7.9	36.2 ± 8.1	0.217
Serum creatinine (mg/dL)	0.86 ± 0.16	0.91 ± 0.17	0.071
CRP (mg/L)	91.6 ± 28.4	94.5 ± 31.4	0.616
D-dimer (ng/mL)	683 ± 541.1	882 ± 598.3	0.232
AST (U/L)	48.7 ± 16.5	50.9 ± 19.7	0.532
ALT (U/L)	42.6 ± 18.4	46.1 ± 17.1	0.298
Pathogen causing super-infection (n, %)			
Acinetobacter spp.	23 (52.3)	37 (51.4)	0.926
Pseudomonas aeruginosa	7 (15.9)	15 (20.8)	0.512
Klebsiella	12 (27.3)	19 (26.4)	0.917
Poly-microbial infections	2 (4.5)	1 (1.4)	0.299
Smoking status (yes, n %)	23 (52.3)	30 (41.7)	0.266
Special therapy for COVID-19 treatment (n, %)			
Steroids	29 (65.9)	38 (52.8)	0.165
Lopinavir/ritonavir	6 (13.6)	9 (12.5)	0.860
Remdesivir	8 (18.2)	21 (29.2)	0.185
Tocilizumab	6 (13.6)	17 (23.6)	0.191

CRP, C-reactive protein; AST, aspartate aminotransferase; ALT, Alanine aminotransferase.

**Table 2:** Outcomes of subjects in the two arms of the study.

Outcome	Colistin Group (n = 44)	Non-Colistin (Control Group) (n = 72)	*p*-Value
Hospitalization duration (days) mean	11.3 ± 3.8	12.8 ± 4.7	0.073
Admission for ICU (yes, %)	14 (31.8)	31 (43.1)	0.228
Duration of ICU stay (days)	9.3 ± 3.1	10.4 ± 3.8	0.108
Mechanical ventilation (yes, %)	10 (22.7)	17 (23.6)	0.913
All-cause-mortality at 30 days (n, %)	14 (31.8)	17 (23.6)	0.332
Microbiological eradication rate (n, %)	35 (79.5)	52 (72.2)	0.377

**Table 3:** Logistic regression of the independent predictors associated with the all-cause mortality at 30 days.

Parameter	Odds Ratio	95% Confidence Interval (CI)
Age (>60)	1.3	0.74–2.5
Chronic respiratory diseases	1.9	1.02–3.9
Colistin use	1.5	0.7–3.5
Nephrotoxicity	2.6	1.1–6.2
Main pathogen		
Acinetobacter baumannii	0.76	0.48–1.2
Pseudomonas aeruginosa	1.4	0.49–3.7
Klebsiella pneumonia	1.7	0.96–3.1

## Data Availability

The datasets generated and/or analyzed during the current study are available from the corresponding author upon reasonable request.

## Supplementary Materials

The following supporting information can be downloaded at: https://www.mdpi.com/article/10.3390/medicina61050884/s1, Table S1: Logistic regression analysis of the independent predictors associated with acute kidney injury in the study population.
