# Supplementary material for: The Efficacy and Tolerability of Colistin Versus Non-Colistin Antimicrobial Regimens Among Hospitalized COVID-19 Patients with Multidrug-Resistant Bacterial Superinfection: An Observational Multicenter Study
Source: Medicina (Kaunas). 2025 May 13;61(5):884. doi: 10.3390/medicina61050884 (PMC12113364; doi:10.3390/medicina61050884)
Supplement: Supplementary file 1 [file medicina-61-00884-s001.zip › medicina-3425473-supplementary.pdf]

Supplementary Table S1: Logistic regression analysis of the independent predictors associated with acute kidney injury in the study population

**Parameter**	**Nephrotoxicity**		
	**OR**	**(95%CIs)**	**P-value**
Group (%)			
IV colistin group	2.86	1.17 – 7.02	**0.021**
Control group	Ref.	Ref	--
Age (>60 years, n (%)	1.08	1.012–1.13	**0.017**
Gender, Male (%)	1.28	0.53 – 3.06	0.586
Co-administered antibiotic therapy (n,%)			
Carbapenem	0.603	0.161 – 2.23	0.446
Piperacillin-tazobactam	1.057	0.347 – 3.21	0.922
Macrolides	0.593	0.173 – 2.04	0.407
Cephalosporins	0.748	0.076 – 7.33	0.803
Fluoroquinolones	Ref.	Ref.	--
Comorbidities (n, %)			
Obesity	Ref	Ref	--
Diabetes mellitus	2.65	1.07 – 6.51	**0.034**
Hypertension	0.640	0.191 – 2.14	0.470
Chronic Respiratory diseases	0.724	0.073 – 7.16	0.783
Pathogen causing super-infection (n, %)			
Acinetobacter spp.	1.35	0.462 – 3.95	0.582
Pseudomonas aeruginosa	0.807	0.229 – 2.84	0.738
Klebsiella	1.174	0.357 – 3.855	0.791
Poly-microbial infections	Ref	Ref	Ref
Smoking status (yes, n %)	0.976	0.407 – 2.34	0.957
Special therapy for COVID-19 treatment (n, %)			
Steroids	Ref	Ref	--
Lopinavir/ritonavir	2.78	0.98 – 7.90	0.056
Remdesivir	2.93	1.15 – 7.463	**0.024**
Tocilizumab	2.57	1.02 – 6.46	**0.045**

Significance expressed as bold
